# Effect of psychological intervention on the quality of life and mental health of leukemia patients: a meta-analysis

**DOI:** 10.3389/fpsyg.2025.1528512

**Published:** 2025-05-09

**Authors:** BingLing Yin, XiaoRong Liu, YanQin Mao, YanQi Han

**Affiliations:** Department of Hematology, The First Affiliated Hospital of Soochow University, Suzhou, China

**Keywords:** psychological intervention, leukemia, mental health, chemotherapy, quality of life

## Abstract

**Background:**

Currently, chemotherapy is the main treatment for leukemia. According to relevant reports, the first remission rate of adult patients after chemotherapy is about 60–70%, and about 1/5 of patients can improve their survival time to 5 years or longer. However, due to the lengthy process of chemotherapy, various adverse reactions may occur, leading to negative emotions such as anxiety, depression, and fear in patients, reducing their compliance with chemotherapy, and posing a great physical and psychological challenge to patients. For the diagnosis and treatment of leukemia, there is a very close relationship between psychological status and treatment effectiveness. A good mentality is beneficial for patients to better cope with the disease and prolong their survival time.

**Methods:**

Retrieve PubMed, Web of Science, Embase, Cochrane Library, CNKI, Wanfang and search for randomized controlled trials (RCTs) related to the treatment of psychological intervention on the quality of life and mental health of leukemia patients published from the establishment of the library until August 2024. The retrieved literature will be independently screened by two researchers, and the methodological quality of the included literature will be evaluated using the bias risk assessment tool recommended in Cochrane 5.1 manual, followed by data statistical analysis.

**Results:**

A total of 1,792 patients were included in 18 studies. The results show the Self-Rating Anxiety Scale of the test group, which was significantly lower (*p* < 0.01, SMD: −6.65; 95% CI: −7.94–−5.35) than the control group. Self-Rating Depression Scale of the test group was lower (*p* < 0.01, SMD: −7.96; 95% CI: −9.52–−6.40). European Organization for Research and Treatment of Cancer Quality of Life Questionnaire Core 30 (*p* < 0.01, SMD: 10.76; 95% CI: 6.27–15.24) of the test group was higher and Pittsburgh Sleep Quality Index (*p* < 0.01, SMD: −3.17; 95% CI: −4.68–−1.67) was lower.

**Conclusion:**

The results indicated that psychological intervention can improve the level of SAS, SDS, QLQ-C30, PSQI in patients with leukemia.

## 1 Introduction

Cancer remains a major global health issue, with tumors causing 9.56 million deaths in 2017, the second leading cause worldwide. Leukemia, a common malignant blood cancer, affects multiple organs and has high incidence and mortality rates (GBD 2017 Causes of Death Collaborators, 2019). In 2020, leukemia caused 310,000 deaths globally, ranking 10th, with China accounting for 60,000 cases, ranking 9th. A 2022 report from China’s National Cancer Registration Center showed a leukemia incidence of 6.21 per 100,000 and a mortality rate of 5.57 per 100,000, ranking 12th and 9th among malignant tumors, respectively (Bray et al., 2020). Leukemia arises from uncontrolled proliferation of immature hematopoietic stem cells, infiltrating various tissues and impairing normal blood cell production (Zheng et al., 2022). It is classified into acute and chronic types: acute leukemia progresses rapidly, with immature cells halting differentiation early, while chronic leukemia progresses slowly, with cells stopping differentiation at later stages (Arber et al., 2017). Acute leukemia cells stop differentiating in the early stages and are generally primitive cells. The disease progresses rapidly and the course of the disease is only a few Months (Heuser et al., 2021). However, the cell differentiation of chronic leukemia stops at the late stage, usually consisting of mature immature cells. The disease progresses slowly and can last for several years (Yeoh et al., 2013; Schuh et al., 2018).

Compared to minors, the responsibilities that adults bear in family and social activities are more important. Diseases cause enormous economic and psychological pressure, and have a negative impact on their treatment and physical and mental recovery. Currently, chemotherapy is the main treatment for leukemia. According to relevant reports, the first remission rate of adult patients after chemotherapy is about 60–70%, and about 1/5 of patients can improve their survival time to 5 years or even longer (Popescu et al., 2020). However, due to the lengthy process of chemotherapy, various adverse reactions may occur, leading to negative emotions such as anxiety, depression, and fear in patients, reducing their compliance with chemotherapy, and posing a great physical and psychological challenge to patients. For the diagnosis and treatment of leukemia, there is a very close relationship between psychological status and treatment effectiveness. A good mentality is beneficial for patients to better cope with the disease and prolong their survival time (Goyal et al., 2018).

With the transformation of the medical field from a single biomedical model to a more comprehensive biopsychosocial model, people are paying more and more attention to patients’ mental health issues, viewing them as a key social issue. A large amount of research has focused on the mental health status of leukemia patients during the treatment process. Research has shown that psychological intervention measures can effectively improve the mental health status and subjective well-being of leukemia patients after chemotherapy (Chacin-Fernández et al., 2019). At present, there are various psychological intervention methods available, including cognitive-behavioral therapy, emotional release therapy, and providing more humanistic care. The main purpose of these intervention measures is to help patients cope with and alleviate their negative emotions. By applying these methods, more comprehensive support can be provided for leukemia patients, helping them maintain a better psychological state when facing disease challenges. It is understood that this is the first summary of the impact of psychological intervention on the quality of life and mental health of leukemia patients.

## 2 Methods

### 2.1 Study design

This meta-analysis encompasses randomized controlled trials investigating the efficacy of psychological intervention on the quality of life and mental health of leukemia patients.

### 2.2 Research participants

Patients with leukemia.

### 2.3 Intervention categories

In the study, individuals with leukemia assigned to the experimental group received psychological intervention as a treatment, whereas those in the control group were administered either a placebo or traditional therapy.

### 2.4 Outcome indicators

Participation in the study necessitates the presence of at least one of the listed outcome indicators. ①Self-Rating Anxiety Scale (SAS); ②Self-Rating Depression Scale (SDS); ③European Organization for Research and Treatment of Cancer Quality of Life Questionnaire Core 30 (QLQ-C30); ④Pittsburgh Sleep Quality Index (PSQI).

### 2.5 Literature retrieval strategy

Database searches were performed in PubMed, Embase, Cochrane Library, Web of Science, CNKI and Wanfang up to August 2024. The search utilized a combination of subject headings and free-text terms, and developed corresponding search strategies based on the different databases. The English keywords utilized were: “psychological intervention,” “leukemia,” and “mental health.”

### 2.6 Literature screening and data extraction

Two investigators conducted a literature screening process independently, adhering to predefined inclusion and exclusion criteria. In cases of disagreement, they resolved discrepancies through discussion and, if necessary, sought arbitration from a third party. The screening process began with a review of titles and abstracts to exclude clearly irrelevant studies, followed by a full-text review to assess eligibility for inclusion. After this initial screening, the final set of included literature was established. Data extraction from the eligible literature was performed independently by two researchers using Excel, with discrepancies addressed through discussion and, if required, consultation with a third party. The data extracted encompassed details such as: principal author, publication year, participant count, gender distribution, mean age, treatment interventions, and outcome measures.

### 2.7 Statistical analysis

In this analysis, Stata software was employed to conduct the meta-analysis. For categorical outcomes, the odds ratio (OR) was utilized to indicate the differences between groups. Whereas for continuous outcomes, either the weighted mean difference (WMD) or the standardized mean difference (SMD) was applied to illustrate group disparities. The findings were presented with a 95% confidence interval (CI), and heterogeneity was assessed using the I2 statistic. The choice between a fixed-effects model (FEM) or a random-effects model (REM) was determined by the degree of heterogeneity observed across studies. Furthermore, Begg’s and Egger’s tests were conducted to assess the presence of publication bias within the study. In instances where significant clinical heterogeneity was identified, sensitivity analysis was performed to identify potential sources of this heterogeneity.

## 3 Results

### 3.1 Literature search results

**Figure 1:**
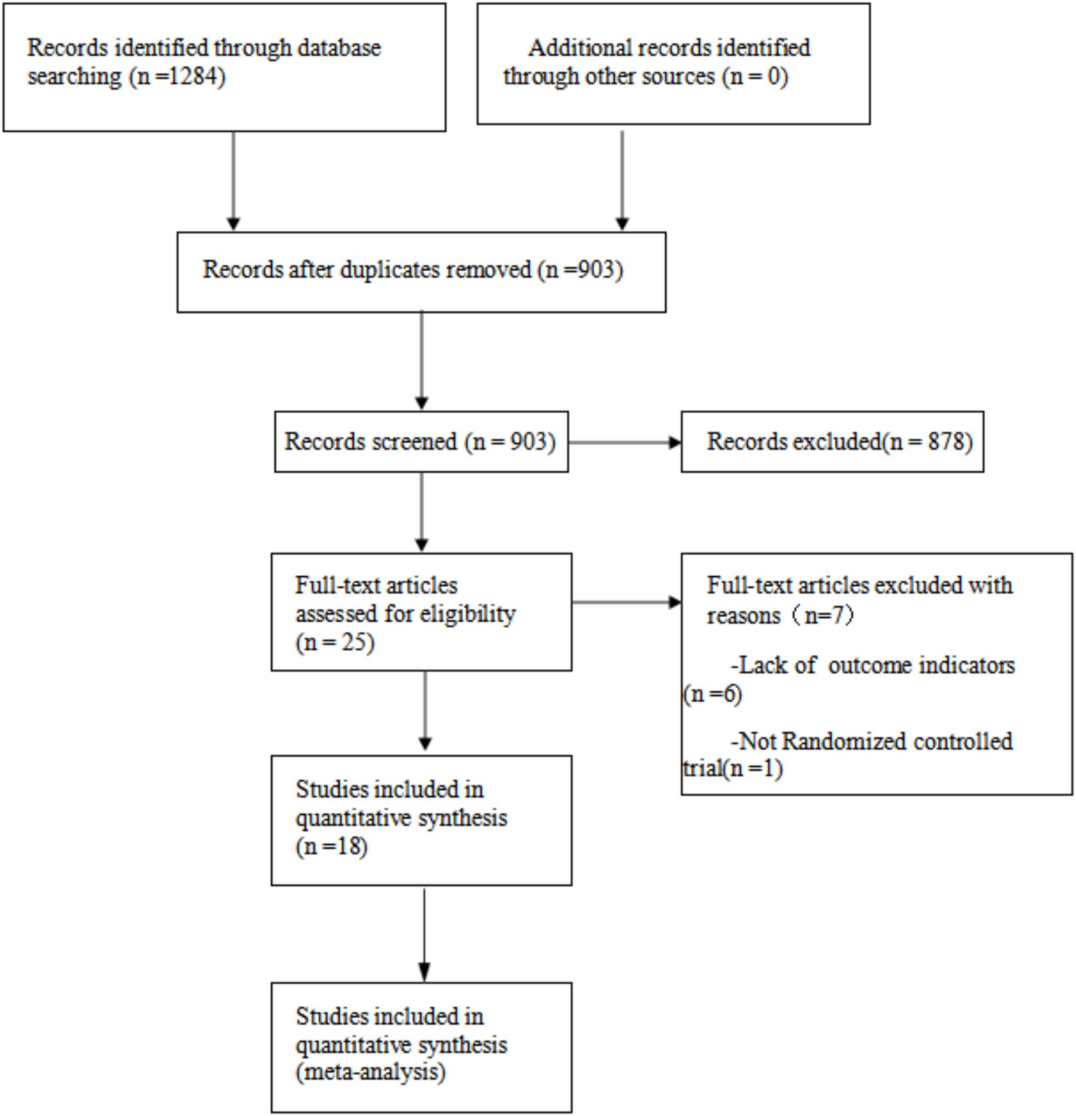
Flow chart.

**Figure 2:**
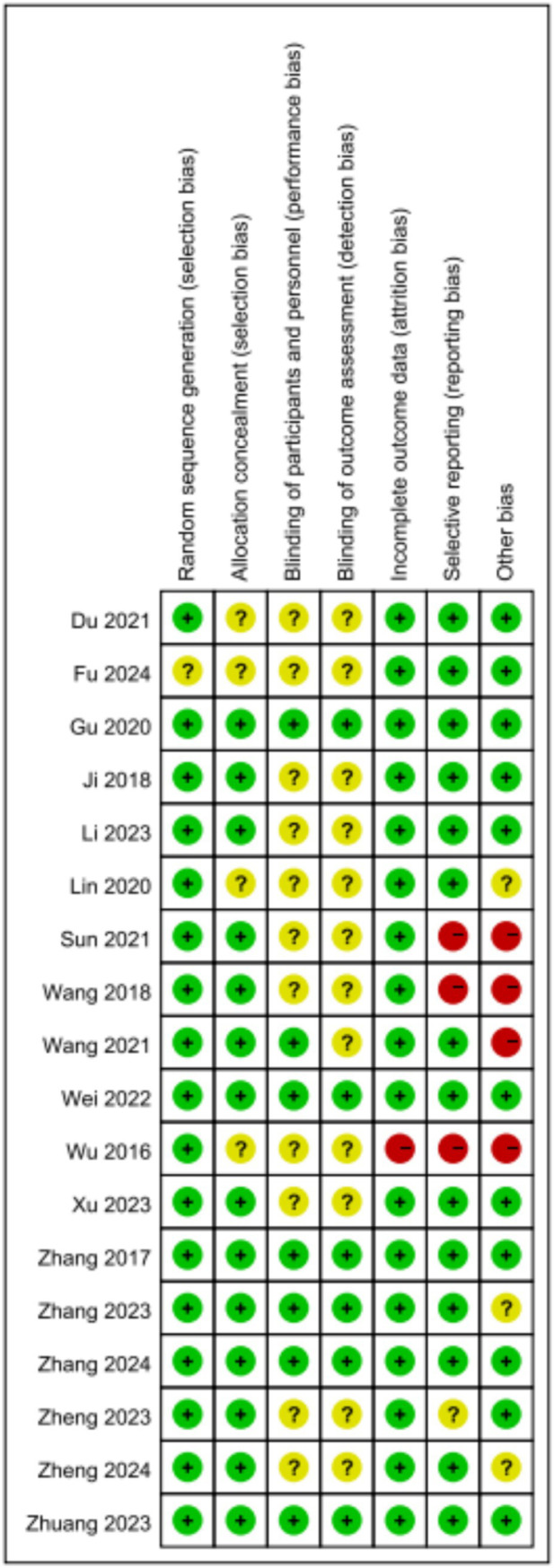
Risk of bias summary.

**Figure 3:**
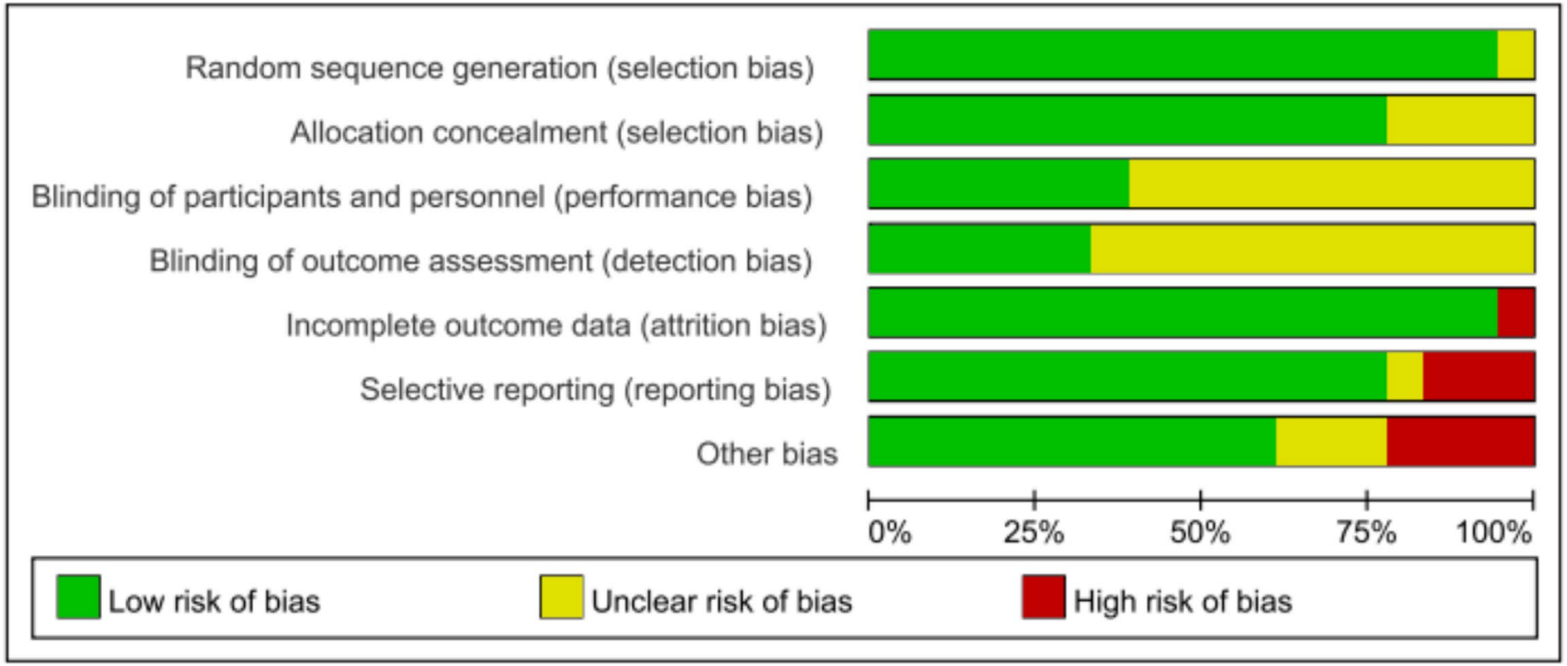
Risk of bias graph.

A total of 1,284 potentially relevant articles were identified through the search process, which resulted in 903 unique records after the removal of duplicates. Following the review of titles and abstracts, 25 articles was selected for further assessment. Subsequent to the exclusion of articles that did not meet the outcome criteria and those that were not randomized controlled trial, a final total of 18 articles were included in the analysis (Zhang et al., 2017; Wei and Li, 2022; Zheng et al., 2024; Fathima et al., 2025; Zheng et al., 2023; Krishnan and Adigopula, 2025; Wang, 2021; Lin, 2020; Musie et al., 2025; Zhuang et al., 2023; Ahmed et al., 2025; Wang et al., 2018; Xu et al., 2023; Kim et al., 2011; Du, 2021; Gu et al., 2020; Li et al., 2023; Ji et al., 2018). The basic characteristics of the included studies are summarized in Table 1, and the PRISMA flow diagram, illustrating the study selection process, is presented in Figure 1. The literature incorporated in this study is of relatively high quality (Figures 2, 3).

**Table 1:** The basic characteristics of the included studies.

References	Cases treat/con	Sex (Male/Female)	Age (years)	Treat group	Con group	Outcomes measures
Zhang et al. (2017)	33/32	36/29	35.64 ± 15.60/33.58 ± 14.75	Psychological intervention	Placebo	①②④
Wei and Li (2022)	45/45	44/46	36.14 ± 10.68/35.74 ± 11.23	Psychological intervention	Placebo	①②
Zheng et al. (2024)	40/40	48/32	46.28 ± 4.26/46.41 ± 4.37	Psychological intervention	Placebo	①②
Fu et al. (2024)	48/48	75/21	42.54 ± 1.08/42.14 ± 1.23	Psychological intervention	Placebo	①②
Zheng et al. (2023)	50/50	52/48	70.1 ± 8.9/69.7 ± 9.3	Psychological intervention	Placebo	①②
Zhang (2023)	94/95	94/95	40.61 ± 1.22/40.52 ± 1.21	Psychological intervention	Placebo	①②
Wang (2021)	51/51	54/48	37.9 ± 12.4/37.4 ± 12.1	Psychological intervention	Placebo	①②
Lin (2020)	46/46	49/43	43.7 ± 11.8/44.2 ± 12.1	Psychological intervention	Placebo	①②
Wu (2016)	32/32	35/29	43.51 ± 2.27/42.57 ± 2.19	Psychological intervention	Placebo	①②
Zhuang et al. (2023)	52/54	63/43	56.82 ± 3.43/56.27 ± 3.25	Psychological intervention	Placebo	①②④
Wang (2021)	34/34	35/33	42.7 ± 10.6/41.2 ± 10.3	Psychological intervention	Placebo	①②
Wang et al. (2018)	42/42	46/38	41.4 ± 8.4/41.7 ± 9.1	Psychological intervention	Placebo	①②
Xu et al. (2023)	55/55	60/50	45.98 ± 5.54/46.12 ± 4.11	Psychological intervention	Placebo	③
Zhang et al. (2024)	50/50	60/40	45.07 ± 6.49/42.69 ± 5.82	Psychological intervention	Placebo	③
Du (2021)	46/46	47/45	26.94 ± 8.05/27.63 ± 7.47	Psychological intervention	Placebo	③
Gu et al. (2020)	48/48	56/40	18–65	Psychological intervention	Placebo	①②④
Li et al. (2023)	80/80	90/70	41.22 ± 7.17/41.15 ± 7.25	Psychological intervention	Placebo	①②④
Ji et al. (2018)	49/49	53/45	57.38 ± 4.93/59.04 ± 5.02	Psychological intervention	Placebo	①②③

Treat, treatment; Con, control. ① Self-Rating Anxiety Scale (SAS); ② Self-Rating Depression Scale (SDS); ③ European Organization for Research and Treatment of Cancer Quality of Life Questionnaire Core 30 (QLQ-C30); ④ Pittsburgh Sleep Quality Index (PSQI).

### 3.2 Self-rating Anxiety Scale (SAS)

**Figure 4:**
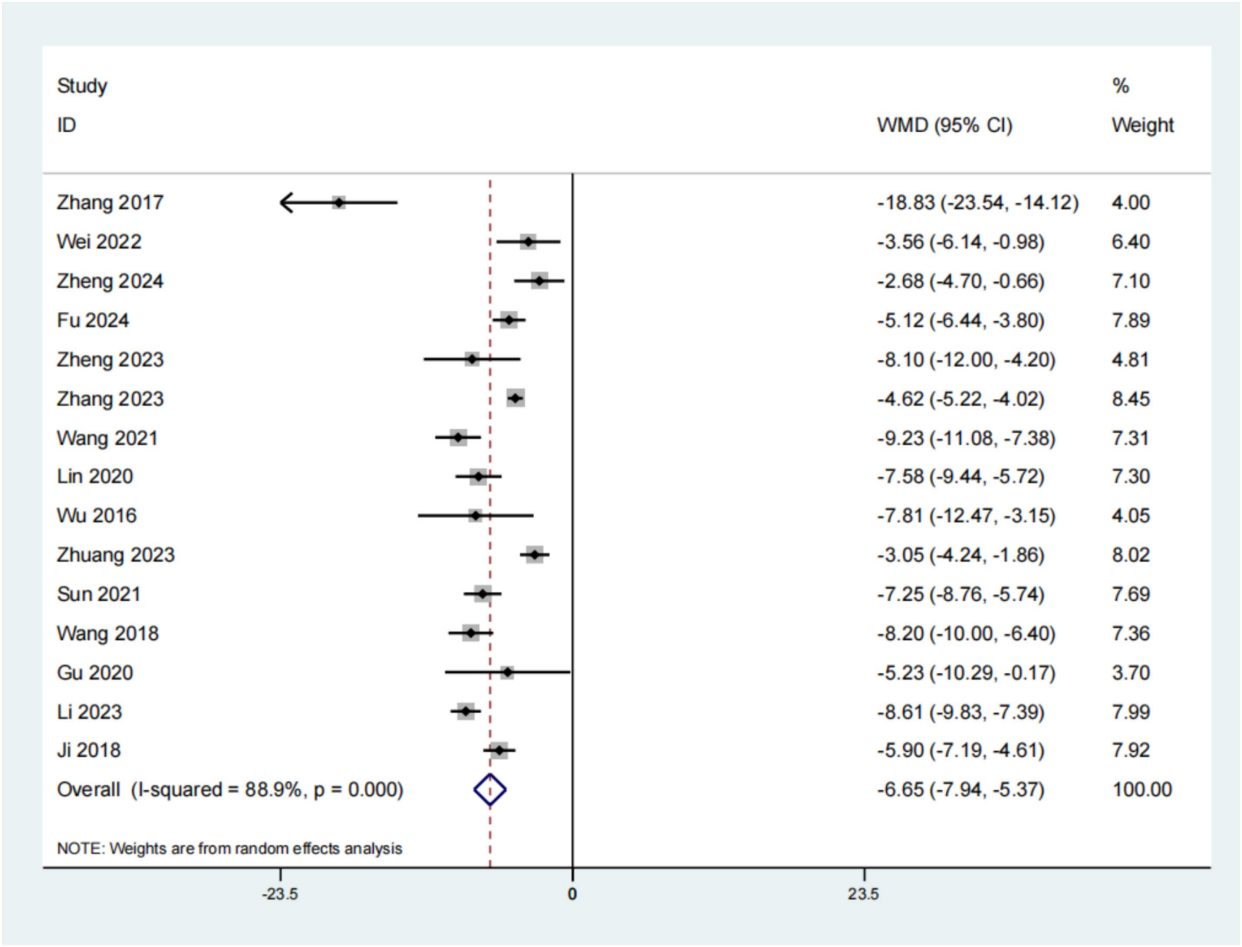
Forest plot of SAS.

A cumulative total of 15 studies have reported on the impact of psychological intervention on SAS in patients with leukemia. The findings indicated a statistically significant improvement (*p* < 0.01, SMD: −6.65; 95% CI: −7.94–−5.35; Figure 4). In this analysis, Begg’s and Egger’s tests were utilized to assess the potential for publication bias concerning the reported outcome measures. The results of the Begg’s test were non-significant with *p* = 0.488, exceeding the 0.05 threshold, and similarly, the Egger’s test also yielded non-significant results with *p* = 0.088. These findings suggest that there is no evidence of publication bias in the study. This suggests that the SAS outcomes for individuals with leukemia who received psychological intervention were superior to those in the placebo group.

### 3.3 Self-rating Depression Scale (SDS)

**Figure 5:**
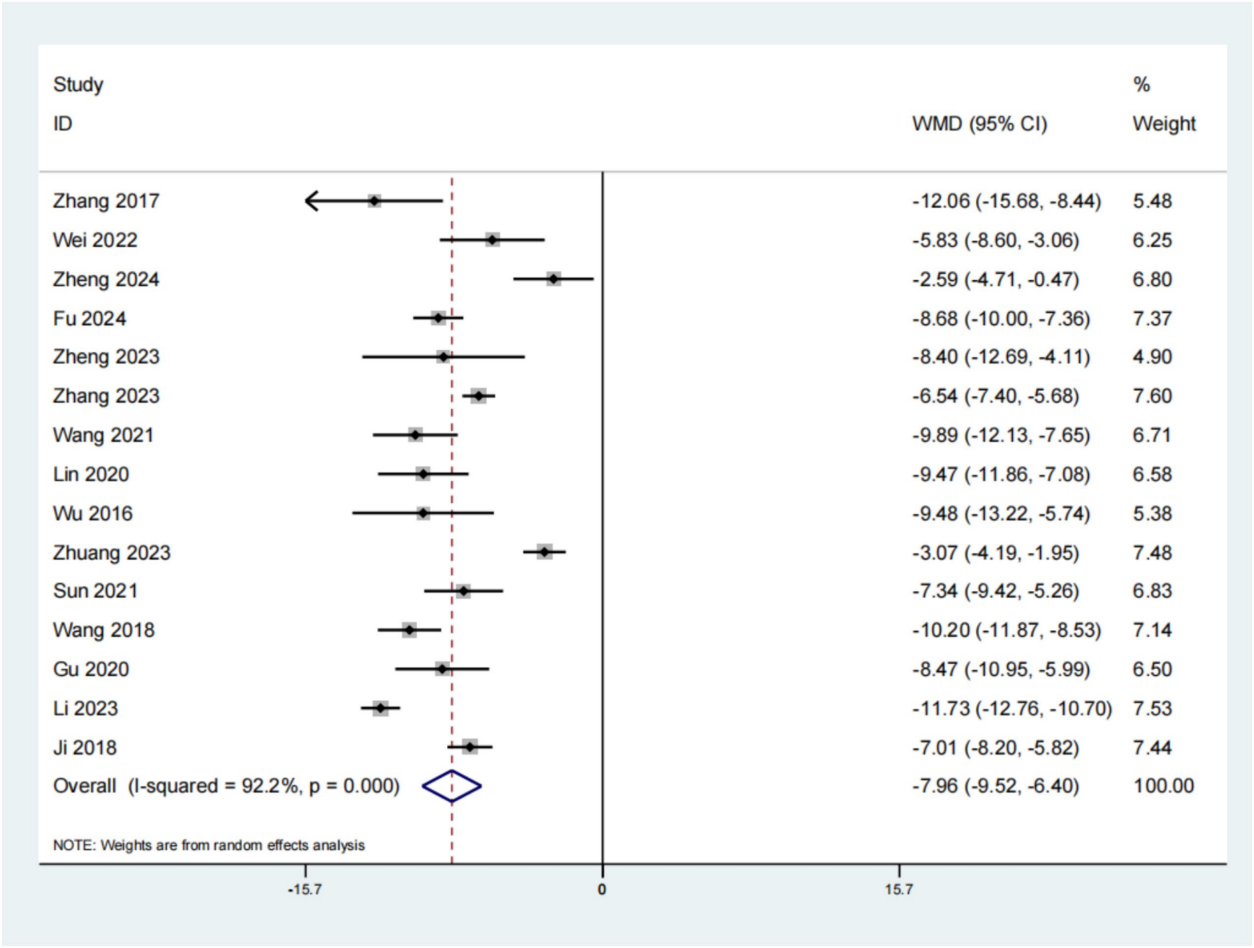
Forest plot of SDS.

A cumulative total of 15 studies have reported on the impact of psychological intervention on SDS in patients with leukemia. The findings indicated a statistically significant improvement (*p* < 0.01, SMD: −7.96; 95% CI: −9.52–−6.40; Figure 5). The results of the Begg’s test were non-significant with *p* = 0.767, exceeding the 0.05 threshold, and similarly, the Egger’s test also yielded non-significant results with *p* = 0.634. These findings suggest that there is no evidence of publication bias in the study. This suggests that the SDS outcomes for individuals with leukemia who received psychological intervention were superior to those in the placebo group.

### 3.4 European organization for research and treatment of cancer Quality of Life Questionnaire Core 30 (QLQ-C30)

**Figure 6:**
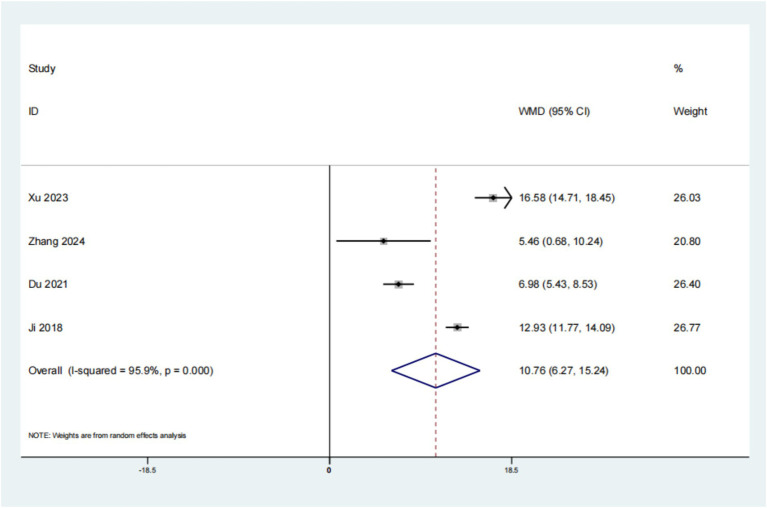
Forest plot of QLQ-C30.

A cumulative total of 4 studies have reported on the impact of psychological intervention on QLQ-C30 in patients with leukemia. The findings indicated a statistically significant improvement (*p* < 0.01, SMD: 10.76; 95% CI: 6.27–15.24; Figure 6). The results of the Begg’s test were non-significant with *p* = 1.000, exceeding the 0.05 threshold, and similarly, the Egger’s test also yielded non-significant results with *p* = 0.764. These findings suggest that there is no evidence of publication bias in the study. This suggests that the QLQ-C30 outcomes for individuals with leukemia who received psychological intervention were superior to those in the placebo group.

### 3.5 Pittsburgh Sleep Quality Index (PSQI)

**Figure 7:**
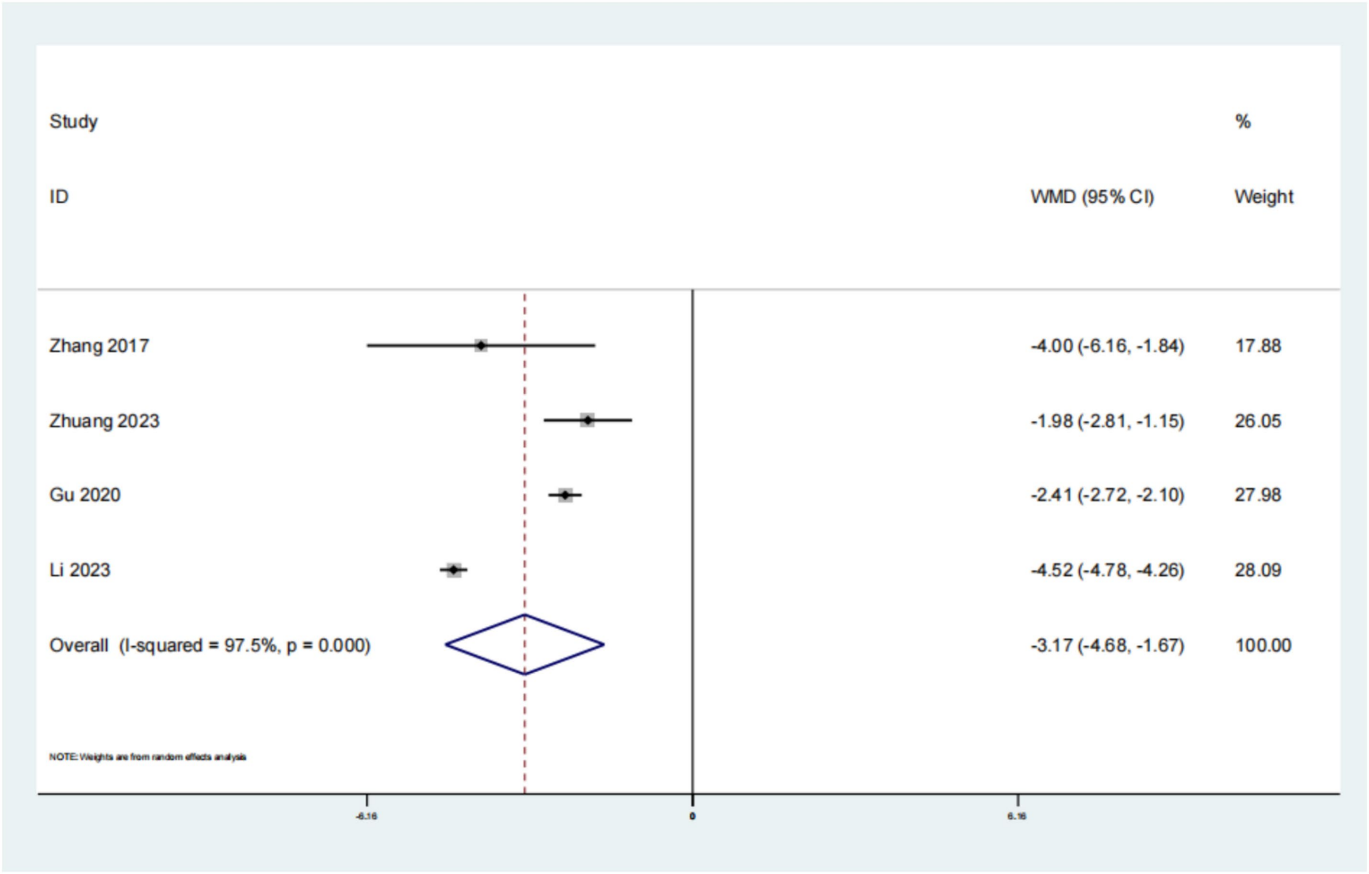
Forest plot of PSQI of denosumab in treatment of osteoporosis in patients with rheumatoid arthritis.

A cumulative total of 4 studies have reported on the impact of psychological intervention on PSQI in patients with leukemia. The findings indicated a statistically significant improvement (*p* < 0.01, SMD: −3.17; 95% CI: −4.68–−1.67; Figure 7). The results of the Begg’s test were non-significant with *p* = 1.000, exceeding the 0.05 threshold, and similarly, the Egger’s test also yielded non-significant results with *p* = 0.704. These findings suggest that there is no evidence of publication bias in the study. This suggests that the PSQI outcomes for individuals with leukemia who received psychological intervention were superior to those in the placebo group.

## 4 Discussion

Following the screening process, 1792 participants across 18 studies were considered, with 895 allocated to the experimental group and 897 to the control group. The analysis focused on 15 publications that examined the SAS in individuals with leukemia. The findings revealed a significant difference (*p* < 0.01, SMD: −6.65; 95% CI: −7.94–−5.35), suggesting that the administration of psychological intervention for leukemia in this patient population resulted in superior SAS outcomes compared to conventional treatment approaches. This intervention not only improves patients’ treatment compliance, but also reduces the risk of adverse reactions, thereby improving their quality of life. In addition, personalized health education can significantly improve the anxiety of leukemia patients. By providing customized health information and psychological support to patients, we help them better understand their disease status, master strategies to deal with potential adverse reactions during treatment, thereby reducing SAS scores and improving life satisfaction. The implementation of psychological intervention, whether it is family centered intervention or individualized health education, has shown a positive impact on the mental health of leukemia patients and their families. These intervention measures effectively alleviate patients’ anxiety and depression symptoms and improve their psychological state by providing emotional support, enhancing disease awareness, and improving coping abilities. The improvement of this psychological state plays an important role in enhancing treatment effectiveness, improving patients’ quality of life, and overall quality of life. Therefore, medical professionals and mental health workers should attach importance to the application of psychological intervention in the treatment of leukemia, and provide comprehensive and continuous psychological support and nursing services for patients and their families (Albrecht and Bryant, 2019). The analysis focused on 15 publications that examined the SDS in individuals with leukemia. The findings revealed a significant difference (*p* < 0.01, SMD: −7.96; 95% CI: −9.52–−6.40), suggesting that the administration of psychological intervention for leukemia in this patient population resulted in superior SDS outcomes compared to conventional treatment approaches.

The analysis focused on 4 publications that examined the QLQ-C30 in individuals with leukemia. The findings revealed a significant difference (*p* < 0.01, SMD: 10.76; 95% CI: 6.27–15.24), suggesting that the administration of psychological intervention for leukemia in this patient population resulted in superior QLQ-C30 outcomes compared to conventional treatment approaches. The QLQ-C30 score is a tool developed by the European Organization for Research and Treatment of Cancer to assess the quality of life of cancer patients. This scale contains 30 items with scores ranging from 0 to 100, with higher scores indicating higher quality of life. It evaluates multiple dimensions, including physical function, role function, cognitive function, emotional function, social function, symptoms such as fatigue, pain, nausea and vomiting, as well as overall health status and overall quality of life (Lepretre et al., 2021). For leukemia patients, the QLQ-C30 score can help healthcare professionals assess changes in their quality of life during treatment. The analysis focused on 4 publications that examined the PSQI in individuals with leukemia. The findings revealed a significant difference (*p* < 0.01, SMD: −3.17; 95% CI: −4.68–−1.67), suggesting that the administration of psychological intervention for leukemia in this patient population resulted in superior PSQI outcomes compared to conventional treatment approaches. PSQI is a tool used to assess an individual’s sleep quality over the past month. For leukemia patients, sleep quality is an important consideration factor, as the treatment of the disease and related physiological and psychological changes may significantly affect the patient’s sleep. A family centered intervention model can significantly improve the psychological state of leukemia patients, reduce anxiety and depression symptoms, and may indirectly improve sleep quality. In addition, mindfulness relaxation training has been shown to improve cancer-related fatigue and sleep quality in leukemia chemotherapy patients.

## 5 Conclusion

This meta-analysis reviewed 18 randomized controlled trials involving 1,792 leukemia patients to evaluate the effects of psychological interventions. The results showed significant reductions in anxiety and depression levels, as measured by the Self-Rating Anxiety Scale (SAS) and Self-Rating Depression Scale (SDS) (*p* < 0.01). Family-centered psychological interventions and individualized health education emerged as effective approaches, improving patient compliance, reducing emotional distress, and enhancing overall well-being. These findings highlight the importance of integrating psychological support into leukemia treatment to optimize patient outcomes and quality of life.

## Data Availability

The raw data supporting the conclusions of this article will be made available by the authors, without undue reservation.

## References

[ref1] Ahmed F. R. Al-Yateem N. Nejadghaderi S. A. Saifan A. R. Farghaly Abdelaliem S. M. ME A. R. (2025). Harnessing machine learning for predicting successful weaning from mechanical ventilation: a systematic review. Aust. Crit. Care 38:101203. doi: 10.1016/j.aucc.2025.101203, PMID: 40058181

[ref2] Albrecht T. A. Bryant A. L. (2019). Psychological and financial distress Management in Adults with Acute Leukemia. Semin. Oncol. Nurs. 35:150952. doi: 10.1016/j.soncn.2019.150952, PMID: 31753705

[ref3] Arber D. A. Borowitz M. J. Cessna M. Etzell J. Foucar K. Hasserjian R. P.. (2017). Initial diagnostic workup of acute leukemia: guideline from the College of American Pathologists and the American Society of Hematology. Arch. Pathol. Lab Med. 141, 1342–1393. doi: 10.5858/arpa.2016-0504-CP, PMID: 28225303

[ref4] Bray F. Ferlay J. Soerjomataram I. Siegel R. L. Torre L. A. Jemal A. (2020). Global cancer statistics 2018: GLOBOCAN estimates of incidence and mortality worldwide for 36 cancers in 185 countries. CA Cancer J. Clin. 70:313. doi: 10.3322/caac.21609, PMID: 30207593

[ref5] Chacin-Fernández J. Chacin Fuenmayor M. Piñerua-Shuhaibar L. Suarez-Roca H. (2019). Psychological intervention based on psychoneuroimmunology improves clinical evolution, quality of life, and immunity of children with leukemia: a preliminary study. Health Psychol. Open 6:2055102919838902. doi: 10.1177/2055102919838902, PMID: 30967959 PMC6444782

[ref6] Du S. (2021). Analysis of the impact of early warning combined with mental awareness intervention on the quality of life and psychological resilience of leukemia patients with hypertension during chemotherapy. Cardiovas. Dis. Prev. Treat. Know. 11, 29–31.

[ref7] Fathima S. Alsugair A. He R. (2025). Myeloid neoplasms with PHF6 mutations: context-dependent genomic and prognostic characterization in 176 informative cases. Blood Cancer J. 15:28. doi: 10.1038/s41408-025-01231-x, PMID: 40025027 PMC11873042

[ref9001] Fu Y. P. Li H. Fan L. P. (2024). Effects of enhanced psychological nursing intervention on psychological status and quality of life in patients with acute myeloid leukemia. J. Rare Dis. 31, 134–136.

[ref8] GBD 2017 Causes of Death Collaborators (2019). Global, regional, and national age-sex-specific mortality for 282 causes of death in 195 countries and territories, 1980-2017: a systematic analysis for the global burden of disease study 2017. Lancet 393, 1736–1788. doi: 10.1016/S0140-6736(18)32203-7 PMC6227606 30496103

[ref9] Goyal N. G. Maddocks K. J. Johnson A. J. Byrd J. C. Westbrook T. D. Andersen B. L. (2018). Cancer-specific stress and trajectories of psychological and physical functioning in patients with relapsed/refractory chronic lymphocytic leukemia. Ann. Behav. Med. 52, 287–298. doi: 10.1093/abm/kax004, PMID: 30084895 PMC6408323

[ref10] Gu J. Yu Y. Wang R. (2020). The effect of comprehensive nursing intervention on the quality of life, sleep quality, and psychological status of patients with acute lymphoblastic leukemia. Nurs. Res. 34, 3738–3740.

[ref11] Heuser M. Ofran Y. Boissel N. Brunet Mauri S. Craddock C. Janssen J.. (2021). Acute myeloid leukaemia in adult patients: ESMO clinical practice guidelines for diagnosis, treatment and follow-up. Ann. Oncol. 32:821. doi: 10.1016/j.annonc.2021.04.005, PMID: 32171751

[ref12] Ji H. Li C. Cheng C. (2018). The impact of WeChat continuity nursing on the psychological state and quality of life of leukemia patients after discharge. Chin. J. Oncol. Clin. Rehab. 25, 229–232.

[ref13] Kim D.-Y. Lee J.-H. Sym S. J. (2011). A prediction model for complete remission upon reinduction for patients with acute myeloid leukemia after failure of anthracycline and cytarabine standard chemotherapy. Ann. Hematol. 90, 1283–1291. doi: 10.1007/s00277-011-1228-x, PMID: 21484304

[ref14] Krishnan S. Adigopula S. (2025). Dasatinib-induced pulmonary arterial hypertension in chronic myeloid Leukaemia: a case report and literature review. Respirol. Case Rep. 13:e70147. doi: 10.1002/rcr2.70147, PMID: 40065794 PMC11893177

[ref15] Lepretre S. Touboul C. Flinois A. Kutikova L. Giannopoulou C. Makhloufi K.. (2021). Quality of life in adults with acute lymphoblastic leukemia in France: results from a French cross-sectional study. Leuk. Lymphoma 62, 2957–2967. doi: 10.1080/10428194.2021.1941924, PMID: 34162314

[ref16] Li J. Zhang Y. Wang M.. (2023). Application effect of problem oriented specialized nursing intervention in patients with acute lymphocytic leukemia. Chin. Med. J. 20, 168–172.

[ref17] Lin H. (2020). Study on the effect of psychological nursing intervention on compliance behavior of leukemia patients in hematology department. Clin. Res. Trad. Chin. Med. 12, 32–47.

[ref18] Musie M. R. Tagutanazvo O. B. Sepeng N. V. (2025). A scoping review on continuing professional development programs for midwives: optimising management of obstetric emergencies and complications. BMC Med. Educ. 25:296. doi: 10.1186/s12909-025-06830-7 39994769 PMC11849364

[ref19] Popescu B. Sheela S. Thompson J. Grasmeder S. Intrater T. DeStefano C.. (2020). Timed sequential salvage chemotherapy for relapsed or refractory acute myeloid leukemia. Clin. Hematol. Int. 2, 27–31. doi: 10.2991/chi.d.191128.001, PMID: 32190831 PMC7079712

[ref20] Schuh A. H. Parry-Jones N. Appleby N. Bloor A. Dearden C. E. Fegan C.. (2018). Guideline for the treatment of chronic lymphocytic leukaemia: a British Society for Haematology guideline. Br. J. Haematol. 182, 344–359. doi: 10.1111/bjh.15460, PMID: 30009455

[ref9004] Wang C. H. (2021). Application effect of psychological nursing intervention in acute leukemia patients receiving chemotherapy. Chinese Health Food, 110–111.

[ref21] Wang X. (2021). Analysis of the application effect of high quality nursing service concept in the nursing of acute leukemia patients. Clin. Res. Trad. Chin. Med. 13, 144–145.

[ref22] Wang X. Wu M. Wang S. (2018). mHealth supportive care intervention for parents of children with acute lymphoblastic leukemia: quasi-experimental pre- and postdesign study. J. Taishan Med. Coll. 39, 1186–1187. doi: 10.2196/mhealth.9981 PMC6301810 30455166

[ref23] Wei F. Li Q. (2022). Effects of positive mental nursing on the post-traumatic growth, negative emotions, and coping style of patients after chemotherapy for leukemia. Iran. J. Public Health 51, 788–796. doi: 10.18502/ijph.v51i4.9239, PMID: 35936547 PMC9288401

[ref9003] Wu Y. T. (2016). Analysis of the effect of psychological nursing in leukemia care. chinese rural Health, 1.

[ref24] Xu N. Xu D. Lingling Z. (2023). The impact of AIDET communication mode nursing on chemotherapy patients with acute leukemia. Chin. Foreign Med. Res. 21, 98–101.

[ref25] Yeoh A. E. Tan D. Li C. K.. (2013). Management of adult and paediatric acute lymphoblastic leukaemia in Asia: resource-stratified guidelines from the Asian oncology summit 2013. Lancet Oncol. 14, e508–e523. doi: 10.1016/S1470-2045(13)70452-2, PMID: 24176570 PMC4059516

[ref9002] Zhang F. Q. (2023). Effect of holistic nursing on sleep quality in patients with chronic myeloid leukemia. World J. Sleep Med. 10, 939–941.

[ref9005] Zhang Q. M. Jiang H. M. Zheng L. (2024). Application of health education based on behavior change model in leukemia patients receiving chemotherapy. China Medical Herald, 21, 183–186.

[ref26] Zhang R. Yin J. Zhou Y. (2017). Effects of mindfulness-based psychological care on mood and sleep of leukemia patients in chemotherapy. Int. J. Nurs. Sci. 4, 357–361. doi: 10.1016/j.ijnss.2017.07.001, PMID: 31406777 PMC6626179

[ref27] Zheng C. Ran L. Wang Y. (2023). The impact of cognitive psychological nursing on the mood state, compliance behavior, and quality of life of elderly leukemia patients. Chin. For. Med. Res. 21, 115–118.

[ref28] Zheng R. Zhang S. Zeng H. Wang S. Sun K. Chen R.. (2022). Cancer incidence and mortality in China, 2016. J. Natl. Cancer Cent. 2, 1–9. doi: 10.1016/j.jncc.2022.02.002, PMID: 39035212 PMC11256658

[ref29] Zheng Q. Zheng Z. Gao M. (2024). The impact of staged continuous nursing intervention on the prognosis of leukemia patients. Chin. Foreign Med. J. 19, 133–136.

[ref30] Zhuang J. Ma J. Tang Y. (2023). The impact of cox health behavior interaction model on chemotherapy compliance, psychological distress, and sleep quality in leukemia patients. Contemp. Nurse 30, 102–106.

